# Supplementary material for: Predictors of burnout among academic family medicine faculty: Looking back to plan forward
Source: PLoS One. 2026 Apr 6;21(4):e0344702. doi: 10.1371/journal.pone.0344702 (PMC13052844; doi:10.1371/journal.pone.0344702)
Supplement: Attachment — Submitted filename: PONE-D-25-29490 Response to Reviewers.pdf [file pone.0344702.s002.pdf]

December 20, 2025

Gholamheidar Teimori-Boghsani
Academic Editor
PLOS ONE

Dear Dr. Teimori-Boghsani,

We thank you and the Reviewers for your detailed review of our manuscript. Please find below our responses to the Reviewers' comments, which we found helpful for improving our manuscript.

Thank you for considering this revised manuscript.

Sincerely,

Viola Antao

Review Comments to the Author

Reviewer #1:

However, I suggest omitting the final sentence "This study provides an opportunity to look back to plan forward" as it reads more like a slogan than a scientific conclusion.

Response: We have deleted this sentence, both in the manuscript and the abstract.

There are a few minor spelling errors and some illogical use of punctuation, which may have occurred during the conversion from Word to PDF.

Response: We have made the necessary corrections.

Major Concerns:
Data Age: The dataset is 14 years old, raising questions about its relevance to current predictors of burnout among family physicians in Canada. While the authors briefly acknowledge this issue, I believe it deserves more thorough reflection. For example, they could discuss how working conditions have evolved over the past 15 years, including the impact of social media, technological advancements, the integration of AI, and the potential for AI-generated clinical documentation—all of which may influence burnout risk.

Response: We have augmented the Discussion of Strengths and Limitations with a more fulsome comparison to recent studies of burnout among physicians, including the

Commonwealth Fund study (November 2025) and two studies of burnout during the COVID 19 pandemic [lines 272-286].

Tool Relevance: How applicable is the burnout assessment tool today? For instance, Sullivan et al. (2025) found that 78% of healthcare workers' responses fell outside the three dimensions measured by the Maslach Burnout Inventory (MBI), suggesting that the tool may no longer fully capture the modern experience of burnout.
Reference: Sullivan et al. (2025). Healthcare worker burnout: Rethinking the Maslach Burnout Inventory. Psychology, Health & Medicine. https://doi.org/10.1080/13548506.2025.2487949

Response: Thank you for forwarding this recent paper. We now reference it in the Discussion. The tool selected (full MBI) was the most appropriate tool available at the time the research was conducted. It remains the standard to which modifications and alternatives are compared. We agree that future research will need to incorporate advances in the understanding and assessment of burnout.

Minor Concerns:
a. In the introduction, the authors state that 6.5 million Canadians lack access to primary care, and "yet" 100 family residency positions remain unfilled. These two statements seem disconnected. In the context of the entire country, 100 unfilled positions is not a particularly high number.

Response: We agree with the Reviewer, and upon reflection we have removed this sentence as it is not essential to the paper.

b. Line 16: "Among family physicians, the prevalence ranged from 25–60%." It would be helpful to include comparative prevalence rates from other medical specialties for context.

Response: We have amended this sentence for clarity and to focus on family physicians.

Reviewer #2:

MS Id: PONE-D-25-29490
Title: Predictors of Burnout Among Academic Family Medicine Faculty: Looking Back to Plan Forward
Type: Research Article
Here are my recommendations about the mentioned MS:
Title:
• Looks good.
Abstract:
• Write subsections of method in abstract in a structured manner.

Response: The Abstract was already set up as a structured abstract with the following headings (subsections): Objective, Design, Setting, Participants, Main outcome measures, Results, and Conclusions. These headings align with PLOS guidelines.

• Do not use abbreviations or symbols in abstract that did not describe previously.

Response: Abbreviations have now been spelled out.

• Summarize results and conclusions.

Response: The Abstract already contains subsections titled "Results" and "Conclusions" where this information was summarized.

Introduction:
• Using the word "Burnout" frequently make readers confuse you can use adjectives instead of repeating the word "Burnout".

Response: We believe that greater confusion would be caused by using other descriptors of burnout. For example, we already highlighted and referenced a concern in the Discussion section that the variability in burnout prevalence estimates may be due in part to inconsistent and unclear use of the term burnout.

Methodology:
• Period, population, and sampling method have to be mentioned in the method section.

Response: The time frame and sampling method were already included in the Methods section. The Setting and Questionnaire sections both state the study was conducted in 2011 among all DFCM faculty. To help clarify this, we have added "1029" to the first sentence in the Questionnaire section as follows: "…we conducted a web-based survey of all 1029 DFCM faculty…"

• Dedicate a subsection for describing study variables, and determining the sample size.

Response: The final sentence in the Questionnaire section stated…The questionnaire content, development, survey promotion, and implementation have been described in detail elsewhere[23]. This reference provides in-depth information on all study variables (questionnaire content). Sample size was also mentioned in this section as being 1029 (the complete list of all DFCM faculty). Our approach is consistent with the guidance to "present and discuss findings concisely"

• The tool's reliability has to be existed.

Response: We used the Maslach Burnout Inventory as the outcome measure and a reference to this was provided in the first sentence of the Outcome measure section.

• The pilot study and the reliability process for the tools have not been mentioned.

Response: The reference cited in the previous comment (ref 23) also provides, as stated, information about the design, testing, and implementation of the survey.

• How do you determine cut-off points? Please explain it.

Response: The citation to the Maslach Burnout Inventory paper describes specific cut-points used for determination of low/medium/high. The citation for this was provided (ref 25).

In terms of dichotomizing the outcomes into "high" versus "low + moderate", our primary concern was family physicians who scored “high” on these measures and identifying predictors for "high" scores. An added benefit is that dichotomized outcomes would also allow for clearer interpretation of the analyses, particularly for the logistic regression analyses.

• Normality distribution of the data

Response: Our data were categorical so this is not applicable.

and the software which used for analyzing the data have to be mentioned.

Response: We have added the following sentence at the end of the Statistical Analysis section: “SPSS version 27 was used for all analyses.”

Results:
• Provide a table for presenting sociodemographic characteristics of the participants.

Response: The Results starts with the heading “Participant Characteristics” which presents this information in text format. Including it again in Table format would be redundant.

• Line 85-88 have to be moved to the method.

Response: The “Statistical Analysis” section in the Methods section outlines the proposed procedures. The “Bivariate Analysis” section in the Results section (lines referred to above), however, presents the results from the bivariate analyses. One of our findings was that the personal accomplishment (PA) subscale lacked variation in responses (99% scored “high” on PA). We therefore did not conduct any bivariate analyses for PA (as proposed in the Statistical Analysis section) and therefore needed to report this finding in the Results. We believe the wording is appropriate and these lines do not need to be moved.

• Write Comments for all of the tables.

Response: It is unclear what is being asked for (e.g., additional comments in the Results section or greater discussion in the Discussion section). All of the Tables have been referred to in the text of Results section to highlight their content. The Tables were designed to be self-explanatory without having to duplicate or describe the contents under the "Results".

• Write p values in the table 5 and 6.

Response: For logistic regression tables we normally don't include p-values (as the 95% Confidence Intervals show whether or not variables were statistically significant). However, Tables 5 and 6 have been redone to include p-values as requested. In addition, we have added the word "p-values" to the following sentence in the Statistical Analysis section to align with the addition of p-values to Tables 5 and 6…. "We report adjusted odds ratios, p-values, and corresponding 95% confidence intervals."

Discussion:
• Looks good.

Conclusion:
• Looks good.

References:
• Change the reference style to the Vancouver style. *I have changed the references to Vancouver Style*

Figures and tables:
• No figure exists.
